# SH3PXD2B is the critical host factor promoting the entry of mycobacteria

**DOI:** 10.1128/aem.00675-26

**Published:** 2026-07-17

**Authors:** Donghui Liu, Ting Zhang, Yujie Sheng, Zhengzhong Xu, Chengkun Zheng, Yingyu Chen, Aizhen Guo, Xin'an Jiao, Xiang Chen

**Affiliations:** 1 Jiangsu Key Laboratory of Zoonosis, Jiangsu Co-Innovation Center for Prevention and Control of Important Animal Infectious Diseases and Zoonoses, Jiangsu Interdisciplinary Center for Zoonoses and Biosafety, Yangzhou University 38043, Yangzhou, China; 2 Key Laboratory of Prevention and Control of Biological Hazard Factors (Animal Origin) for Agrifood Safety and Quality of Ministry of Agriculture and Rural Affairs, Yangzhou University 38043, Yangzhou, China; 3 National Key Laboratory of Agricultural Microbiology, College of Veterinary Medicine, Huazhong Agricultural University 47895, Wuhan, China; Centers for Disease Control and Prevention, Atlanta, Georgia, USA

**Keywords:** *Mycobacterium bovis*, bovine tuberculosis, SH3PXD2B, genome-wide CRISPR screen

## Abstract

Bovine tuberculosis (bTB), caused by *Mycobacterium bovis*, causes major economic losses in the global livestock industry and threatens public health. Macrophages are essential for pathogen clearance, yet serve as the primary intracellular niche for mycobacterial survival. These characteristics emphasize the importance of identifying host factors that regulate macrophage functions during infection. To systematically uncover such factors, we constructed a genome-wide CRISPR-KO library in Bomac cells (the bovine macrophage cell line) and performed iterative infection screening under high selective pressure. Here, the host protein SH3PXD2B was identified as a key host factor to promote mycobacterial invasion: SH3PXD2B was significantly enriched in the screening. *M. bovis* infection significantly increases the expression of SH3PXD2B. The phagocytic activity of macrophages is enhanced in an SH3PXD2B-dependent manner, and the high expression of SH3PXD2B inhibits the migration of macrophages, causing macrophages to remain at the site of infection and promoting the entry of more mycobacteria into macrophages to establish infection. Further results indicate that macrophage migration inhibitory factor is positively regulated by SH3PXD2B and plays an important role in SH3PXD2B-mediated macrophage migration and phagocytic activity. These results indicate that SH3PXD2B is a critical host factor manipulated by *M. bovis* to promote infection. It provides a new potential target for genetic breeding against bTB.

**IMPORTANCE:**

There is a complex interaction between *Mycobacterium bovis* and the host, which has an important impact on the occurrence and development of bTB. However, the host gene regulatory network for *M. bovis* infection is still incomplete, and the specific genes that play a key role in the infection process are still unclear. Bovine tuberculosis remains a major economic and public health burden worldwide. This study identifies SH3PXD2B as a novel host factor that is exploited by *M. bovis* to promote macrophage phagocytosis and inhibit migration, thereby facilitating bacterial invasion. These findings reveal a new pathogenic strategy and suggest that SH3PXD2B may represent a potential target for host-directed therapy and genetic breeding against bTB.

## INTRODUCTION

Bovine tuberculosis (bTB) is a chronic zoonotic infectious disease mainly caused by *Mycobacterium bovis*. The global prevalence rate is as high as 9%, causing huge economic losses to animal husbandry every year. *M. bovis*, belonging to the *Mycobacterium tuberculosis* complex, has a complex pathogenic mechanism similar to that of *Mycobacterium tuberculosis* (Mtb) and a wider host range. In addition, *M. bovis* infection is also one of the causes of human tuberculosis, which seriously threatens human health (1, 2). Macrophages, which are the first line of defense in the host immune system, play a dual role in mycobacterial infection. Specifically, they are the key cells for pathogen clearance and also serve as the primary host cells for mycobacteria (3, 4). Therefore, conducting in-depth research into the interaction between macrophages and mycobacteria, with a particular focus on the regulatory mechanisms of host genes during infection, is imperative for elucidating the molecular underpinnings of mycobacterial infection.

It has been demonstrated in prior studies that certain components of Mtb have the capacity to interfere with or manipulate various host cell functions. This interference is necessary for the microbe’s intracellular survival and virulence (5). To a certain extent, these targeted host factors act as accomplices in the infection process. Numerous studies have shown that inhibiting these host factors, which facilitate mycobacterial infection, can enhance host resistance (6, 7). For instance, the histone deacetylase (HDAC) inhibitor phenylbutyrate has been shown to restrict the proliferation of mycobacteria within macrophages (8). Research has demonstrated that the inhibition of elevated PGE2 levels in late-stage tuberculosis has been shown to reduce infection severity (9). These genes that confer host susceptibility to tuberculosis represent promising targets for its detection and breeding for disease resistance. Furthermore, these relevant inhibitors are being developed as significant tools for the host-directed therapy (HDT) in TB. However, the regulatory network of host genes in mycobacterial infection remains incomplete, and the specific genes that play critical roles during infection are still unclear.

In recent years, the potential of whole-genome screening technologies to reveal host-pathogen interactions has been demonstrated (10, 11). By systematically knocking out host genes, it becomes more feasible to identify key genes involved in the infection process. This study constructed a CRISPR-KO library targeting the bovine genome based on Bomac cells (bovine macrophages). Multiple rounds of *M. bovis* infection were performed, and resistant cells were selected based on a cell survival screen. We found that the gene *SH3PXD2B* (encoding SH3 and PX domain-containing protein 2B [SH3PXD2B]), which has been enriched, contributes to mycobacterial infection. This study demonstrates that mycobacteria promote the expression of SH3PXD2B in macrophages. This protein inhibits the migration of macrophages while enhancing their phagocytic capacity. This enables macrophages to incorporate mycobacteria more effectively through phagocytosis. These results provide novel insights into the mechanisms of mycobacterial invasion.

## MATERIALS AND METHODS

### Bacteria, cells, and plasmids

*M. bovis* Bacillus Calmette-Guérin (BCG) Pasteur was grown in Middlebrook 7H9 medium (BD Diagnostics, US) supplemented with 10% albumin-dextrose-catalase (Difco, US). Bacterial titers were determined by counting colony-forming units (CFUs) on 7H10 agar supplemented with 10% oleic acid-albumin-dextrose-catalase (Difco, US). Bomac cells, RAW264.7 cells, and HEK293T cells were cultured in Dulbecco’s Modified Eagle Medium (DMEM; Gibco, US) supplemented with 10% fetal bovine serum (FBS). THP-1 cells were cultured in Roswell Park Memorial Institute 1640 medium supplemented with 10% FBS. Plasmid pCMV-Myc is stored in our laboratory. To construct plasmids pCMV-SH3PXD2B, bovine *SH3PXD2B* was amplified from cDNA and subcloned into the pCMV-Myc.

### Antibodies

The following antibodies were used in this study: anti-CRISPR-Cas9 antibody (7A9-3A3, Abcam, UK), rabbit anti-SH3PXD2B polyclonal antibody (C-Term; abs113628, Absin, China), Myc tag mouse monoclonal antibody (AF0033, Beyotime, China), HA tag mouse monoclonal antibody (AF5057, Beyotime, China), β-Actin antibody (A2-F6, HUABio, China), horseradish peroxidase (HRP)-conjugated goat anti-mouse IgG (H + L) (AS003, ABclonal, China), and HRP-conjugated goat anti-rabbit IgG (H + L; AS014, ABclonal, China).

### SH3PXD2B overexpression and siRNA interference

Cells were plated in 24-well plates and grown to 80% confluence. For bovine SH3PXD2B overexpression, cells were transfected with 800 ng pCMV-SH3PXD2B or pCMV-Myc (empty vector, EV) using Lip2000 Transfection Reagent (Biosharp, China) according to the manufacturer’s instructions for operation. For siRNA interference, the siRNA-SH3PXD2B or negative control siRNA (siRNA-NC, a scrambled sequence without a specific target) was transfected with Lip2000 Transfection Reagent. The siRNA sequences used are listed in Table S2. The siRNAs were synthesized by Angel Biotech (Suzhou, China).

### Genome-wide CRISPR-Cas9 knockout screen

**Fig 1:**
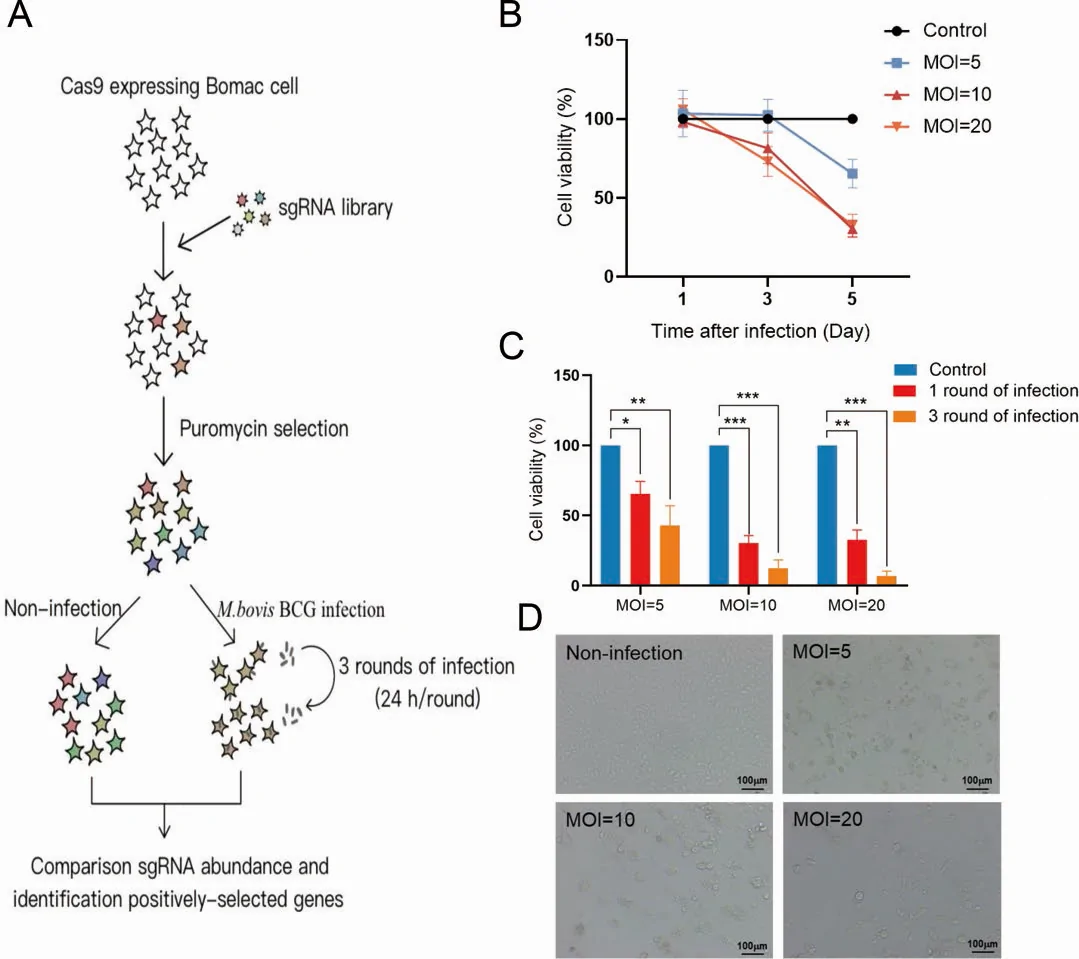
Construction and screening of whole-genome knockout libraries of Bomac. (**A**) Strategy for preparing CRISPR libraries and performing screens. (**B**) Cell survival of Bomac after *M. bovis* BCG infection at different MOIs (**C**) Cell survival of Bomac after multiple rounds of *M. bovis* BCG infection (day 5). (**D**) Cells after multiple rounds of *M. bovis* BCG.

The screening workflow is illustrated in Fig. 1A. First, Cas9-expressing Bomac cells (Bomac-Cas9) were generated by transducing a Cas9 coding construct LentiCas9-Blast (Addgene #52962) and subsequently expanded in the presence of 10 μg/mL blasticidin (12). Based on the bovine genome (ARS-UCD1.3), we designed the sgRNA library targeting all protein-coding genes in the bovine genome (three sgRNAs per gene). The sgRNA fragment library was synthesized by SYNBIO (Suzhou, China) and inserted into the vector plasmid lentiGuide-Puro (Addgene #52963). Subsequently, a high-titer lentiviral library was packaged in HEK293T cells (10). The Bomac-cas9 stable cells were then transduced with the bovine sgRNA pooled library at low multiplicity of infection (MOI) (0.3) to limit co-transduction. The transduced cells were selected with puromycin (1 μg/mL) to generate a knockout cell library. Then, *M. bovis* BCG (MOI = 20) was used for a total of three successive rounds of infection (10). After infection, *M. bovis* BCG-infected host cells were washed three times with PBS and resuspended in complete DMEM containing gentamicin (50 μg/mL) for 2 h to kill extracellular bacteria, washed, and maintained for the rest of the experiments. Genomic DNA was extracted from the surviving cells. The sgRNA sequences were amplified using 2× Phanta MAX Master MIX (Vazyme, China) to prepare the sequencing library (Table 1), and next-generation sequencing was carried out by Sangon Biotech (Shanghai, China). The MAGeCK (version 0.5.9) algorithm was used to identify statistically significant hits (13).

**TABLE 1:** Primer sequences used in this study

Primer name	Sequence (5′−3′)
lib-F	ATGGACTATCATATGCTTACCGTAACTT
lib-R	TGTCTCAAGATCTAGTTACGCCAAG
qβactin-F	CAGCAAGCAGGAGTACGATGAG
qβactin-R	AAGGGTGTAACGCAGCTAACAGT
qMIF-F	ACAGCATCGGCAAGATCGG
qMIF-R	ATCCTGTCCGGGCTAATGC
qpcr-SH3PXD2B-F	GCAATGGAAAACAGCTCGGG
qpcr-SH3PXD2B-R	GATCTCCTCGTAGCCTGCAC
SH3PXD2B-F	TGGCCATGGAGGCCCGAATTCGGATGCCGCCGCGGCGCAGC
SH3PXD2B-R	CCGCGGCCGCGGTACCTCGAGCTAGGGCTTCTTTCTGAGATAGTTGG

### Cloning and analysis of the bovine *SH3PXD2B*

Total RNA was extracted from the Bomac cells using the FastPure Cell/Tissue Total RNA Isolation Kit (Vazyme, China), and cDNA was synthesized using HiScript III RT SuperMix for qPCR (+gDNA wiper; Vazyme, Nanjing, China). To clone bovine *SH3PXD2B*, primers were designed based on the predicted bovine *SH3PXD2B* mRNA sequence (GenBank number XM_024981427.2) (Table 1). The cloned insert was sequenced by Sangon Biotech (Shanghai, China). The amino acid sequence of bovine SH3PXD2B was aligned with orthologs from other species using MEGA-X, and the results were visualized using ESPript (version 3.0).

### BCG infection

Bomac cells were cultured in DMEM supplemented with 10% FBS for 12 h before transfection. Cells were then transfected with 800 ng of pCMV-SH3PXD2B or empty vector, or 20 pmol of siRNA-SH3PXD2B or siRNA-NC (negative control), using Lip2000 transfection reagent. After 24 h, the cells were infected with BCG at the MOI of 10 or 20 for 4 h. Subsequently, the medium was removed, and the cells were washed three times with PBS. Then cells were maintained in complete DMEM containing gentamicin. At selected time points, Bomac cells were lysed in 500 µL PBS containing 1% (vol/vol) Triton X-100. Tenfold serial dilutions of the lysate were performed, followed by plating on 7H10 agar plates to calculate the intracellular bacterial CFU. Cell viability was measured using the CCK8 kit (Beyotime, China) according to the instructions.

### Real-time qPCR assay

Total RNA was extracted from the Bomac cells using the FastPure Cell/Tissue Total RNA Isolation Kit (Vazyme, China), and cDNA was synthesized using the HiScript III RT SuperMix for qPCR (+gDNA wiper). The synthesized cDNA was stored at −20°C until further use. qPCR was performed using Universal SYBR qPCR Master Mix (Vazyme, China) and run for 40 cycles on a BIO-Rad iQ6 instrument. The primers used are listed in Table 1 and Table S1. The mRNA levels were normalized to *β-actin* using the 2^−ΔΔCt^ method.

### Western blotting

Proteins were extracted from cells by lysis with ice-cold RIPA buffer supplemented with a protease inhibitor cocktail (Beyotime, China). Equal amounts of protein per sample were run on an SDS-PAGE and transferred to nitrocellulose membranes (PALL, US). The membranes were blocked with 5% skim milk for 1 h at room temperature and then incubated overnight at 4°C with the primary antibodies. The membranes were washed with TBST and incubated with the appropriate HRP-conjugated secondary antibodies for 2 h at room temperature. After membranes were washed three times, images of protein blots were obtained with a ChemiDoc XRS (Bio-Rad, Marnes-la-Coquette, France) using the Western ECL Substrate kit (Vazyme, China). The density of each band was normalized to that of β-Actin.

### Cell migration assay

Scratch assay: The Bomac cells were seeded into six-well plates and subsequently subjected to transfection with siRNA or plasmids. Following this, the cells were incubated at 37°C with 5% CO₂ to more than 90% confluence. The medium was substituted with serum-free medium. Thereafter, linear scratches were made along a straightedge using a 200 μL pipette tip. Scratches were then gently washed three times with PBS to remove detached cellular debris. Then, cells were incubated in serum-free culture containing gentamicin at 37°C with 5% CO_2_ to allow for wound closure. Microscopic images were captured at various time points after scratching. Wound closure was quantified using ImageJ software (version 1.51).

Transwell assay: Cells transfected with siRNA or plasmid for 24 h were digested with trypsin, then resuspended in DMEM without FBS and adjusted to a cell density of 1 × 10^6^ cells/mL. A total of 100 μL of cell suspension was added to the upper chamber of each well, and 500 μL of complete DMEM was added to the lower chamber. The cells were then incubated at 37°C with 5% CO_2_ for 24 h. The old medium was discarded, and the cells were gently washed three times with sterile PBS. Subsequently, an appropriate amount of 4% paraformaldehyde was added, and the cells were left to stand at room temperature for 5 min to fix them. After fixation, the cells were gently washed three times with PBS, and then 0.1% crystal violet was added and left to stand at room temperature for 15 min for staining. Following staining, the chamber was gently washed with PBS, and the upper layer of cells was gently wiped off with a cotton swab. After drying, the cells were observed under a microscope. All experiments were performed with three independent biological replicates.

### FITC-Dextran uptake assay

The Bomac cells were seeded into 24-well plates and subsequently subjected to transfection with siRNA or plasmids. At least 24 h after transfection, then add 1 mg/mL FITC-Dextran (dilution with serum-free medium); add an equal volume of buffer to the control group; incubate at 37°C for 2 h; wash the cells three times with PBS, allowing each wash to stand for 1 min; and subsequently, detect the fluorescence intensity of the cells using a fluorescence microplate reader. All experiments were performed with three independent biological replicates.

### Statistical analysis

Data represent the mean of a minimum of three independent experiments, with error bars denoting the standard deviation. Student’s *t*-test was used for comparisons between two groups, while one-way analysis of variance was used for multiple-group comparisons. Statistical significance was defined as follows: ns, *P* > 0.05; *, *P* < 0.05; **, *P* < 0.01; ***, *P* < 0.001.

## RESULTS

### Whole-genome screening reveals critical genes associated with mycobacterial infection

In the present study, a screening strategy was employed (Fig. 1A), in which a Bomac cell line was engineered to stably express the Cas9 protein without compromising cell viability (Fig. S1). Based on the bovine genome (ARS-UCD1.3), we designed 62,952 sgRNAs targeting 20,984 protein-coding genes in the bovine genome (three sgRNAs per gene) and established the CRISPR-KO library through puromycin selection. For Bomac cells, infection with the *M. bovis* BCG can result in cell death (Fig. 1B). In order to enhance the screening process and eliminate the occurrence of false positives, a methodical approach was employed. This involved subjecting the Bomac cells to three successive rounds of BCG infection. The procedure resulted in the eradication of over 90% of the cells after 5 days (Fig. 1C and D). This screening period was deemed sufficient for the purposes of this study.

**Fig 2:**
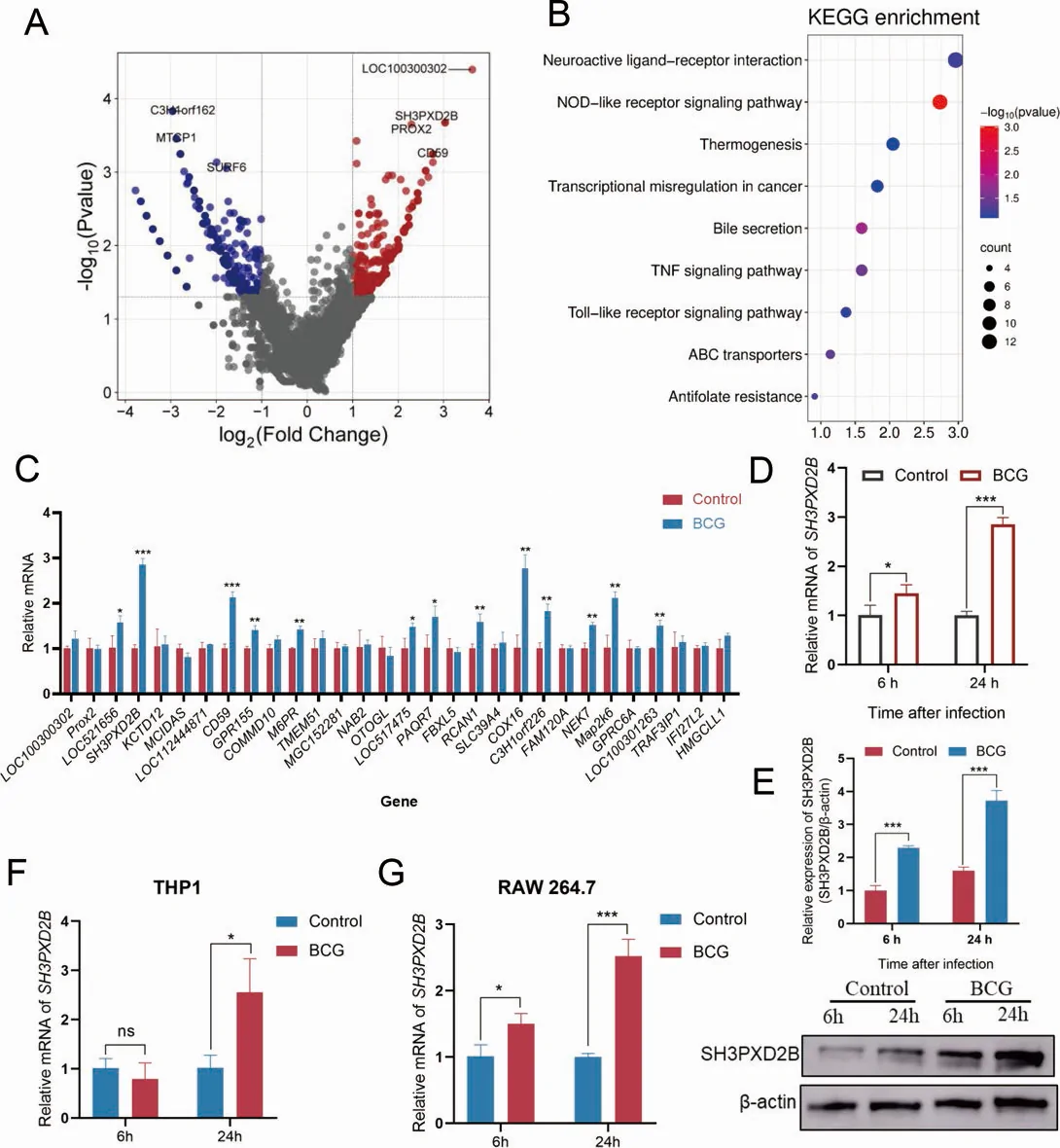
CRISPR screen hit gene *SH3PXD2B* expression is upregulated after BCG infection. (**A**) Volcano plot for CRISPR screen. For each sgRNA target gene, the x-axis indicates its enrichment or depletion after infection, and the y-axis indicates statistical significance as measured by *P* value. Positive and negative screen hits are labeled as red and blue dots. (**B**) Results of KEGG enrichment analysis of positive hit genes. (**C**) The mRNA levels of the top positive hit genes in Bomac after *M. bovis* BCG infection. (**D**) The mRNA levels of *SH3PXD2B* in Bomac after *M. bovis* BCG infection. (**E**) Expression of SH3PXD2B in Bomac after *M. bovis* BCG infection. (**F**) The mRNA levels of *SH3PXD2B* in THP-1 cells. (**G**) The mRNA levels of *SH3PXD2B* in RAW264.7 cells.

To analyze the sequencing results, the MAGeCK program (13) was employed, and genes with log_2_ FC absolute values greater than 1 and *P* < 0.05 were designated as hits (Fig. 2A). The functional annotation clustering was performed using the DAVID bioinformatics database (Fig. 2B). KEGG analysis revealed that positive hit genes (i.e., genes that increased host cell survival) were predominantly enriched in pathways such as NOD-like receptor signaling, Toll-like receptor signaling, TNF signaling, ABC transporters, and cellular antioxidant activity (Fig. 2B).

In the results, some factors that have been enriched, such as HDAC, Toll-like receptor 3 (TLR3), and mitogen-activated protein kinase 12 (MAPK12) have been experimentally validated to be associated with mycobacterial infection (8, 14) (15) (16). Research has demonstrated that the HDAC inhibitor phenylbutyrate exerts a bacteriostatic effect on Mtb in culture, thereby constraining its intracellular growth in macrophages (8). Knockout of HDAC1 in human macrophage lines reduces the intracellular survival of Mtb (14). Furthermore, an increase in Th1 cell numbers in the spleen and a reduction in bacterial burden and tissue damage in the lungs were observed in *Tlr3*^−/−^ mice infected with *M. bovis* BCG (15). Inhibition of p38 MAPK has been demonstrated to suppress the growth of Mtb in mouse macrophages (16). In addition, MAP2K6 was identified in another genome-wide RNA interference screening study of THP-1 cells. They found that MAP2K6 may affect the survival and replication of mycobacteria in macrophages by regulating redox balance and energy metabolism (17). These findings collectively indicate that our screening method is effective.

### Mycobacteria regulate the SH3PXD2B in host cells

**Fig 3:**
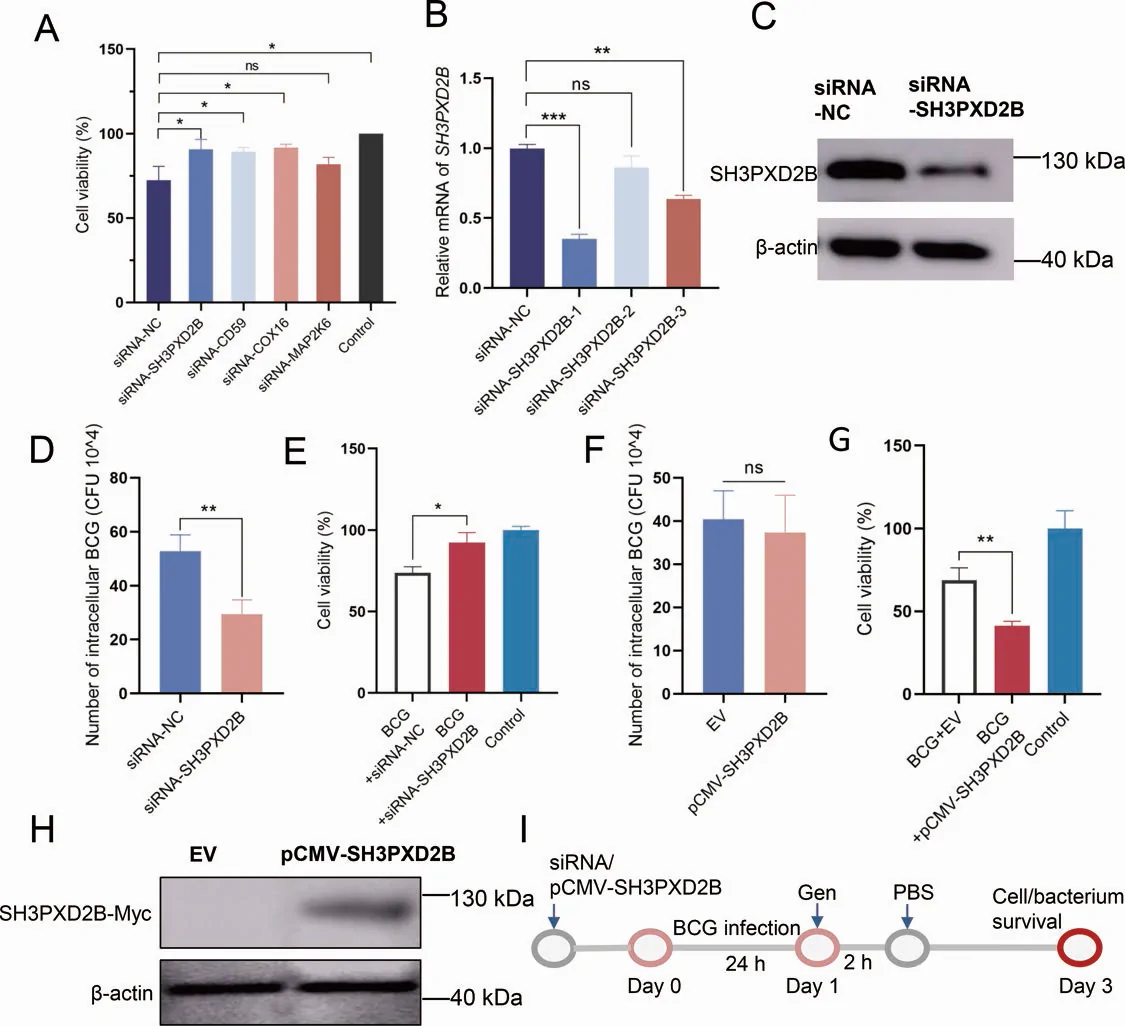
Knockdown of *SH3PXD2B* contributes to host cell survival after BCG infection. (**A**) Cell survival after infection of single knockdown Bomac cells. (**B**) The mRNA levels of *SH3PXD2B* in Bomac after siRNA transfection. (**C**) Expression of SH3PXD2B in Bomac after siRNA transfection. (**D**) Intracellular *M. bovis* BCG levels after infection of Bomac cells with SH3PXD2B knockdown (day 5). (**E**) Cell survival after infection of Bomac cells with SH3PXD2B knockdown (day 5). (**F**) Intracellular *M. bovis* BCG levels in Bomac cells with SH3PXD2B overexpression after BCG infection (day 5). (**G**) Cell survival of Bomac cells with SH3PXD2B overexpression after BCG infection (day 5). (**H**) Expression of SH3PXD2B-Myc in Bomac after pCMV-SH3PXD2B transfection. (**I**) Schematic of siRNA treatment in Bomac cells infected with BCG.

By measuring the transcriptional changes of these positive hit genes during *M. bovis* infection, it was determined that expression of some genes, such as SH3PXD2B and CD59, was significantly upregulated after BCG infection (Fig. 2C). These results suggest that these genes likely respond to BCG infection. A selection of genes was made for further knockdown using siRNA, and it was determined that Bomac cells with knockdown of SH3PXD2B, CD59, or COX16 exhibited significantly increased survival rates under BCG infection conditions (Fig. 3A).

A notable factor is SH3PXD2B, a typical scaffold protein that acts as a key regulator of specific signaling events by binding multiple participants in a signaling pathway (18). It was initially identified as a tyrosine kinase substrate and is also known as tyrosine kinase substrate 4 (TKS4) (19). Mutations in SH3PXD2B are associated with Frank-Ter Haar syndrome, which involves different traits such as skeletal dysplasia, glaucoma, and abnormalities in fat differentiation (20, 21). Meanwhile, SH3PXD2B is found to be highly expressed in a variety of cancer tissues and cells, which promotes the invasion and metastasis of human hepatocellular carcinoma, melanoma, squamous cell carcinoma of the oral cavity, and gastric cancer (22–25).

The bovine SH3PXD2B demonstrated marked upregulation in both RNA transcription and protein expression levels during BCG infection (Fig. 2D and E). Similarly, following the infection of RAW264.7 cells (mouse-derived macrophages) and the THP-1 treated with phorbol ester (human-derived macrophages) with BCG, there were also significant elevations in the transcript levels of *SH3PXD2B* (Fig. 2F and G). Knockdown of SH3PXD2B using siRNA improved cell viability during BCG infection (Fig. 3E). In order to determine whether the alterations in cell viability were associated with the survival of intracellular bacteria, the intracellular survival of BCG was further examined. It was ascertained that the intracellular survival of BCG was considerably diminished in the SH3PXD2B knockdown group compared to the control group (Fig. 3D). Furthermore, when SH3PXD2B was overexpressed using a eukaryotic expression plasmid (Fig. 3H), there was a significant decrease in host cell viability (Fig. 3G). However, there was no significant difference in intracellular bacterial survival (Fig. 3F). This may be due to bacteria escaping from dead cells being overlooked during counting. However, this explanation remains speculative and requires further rigorous verification.

### Sequence analysis of SH3PXD2B

**Fig 4:**
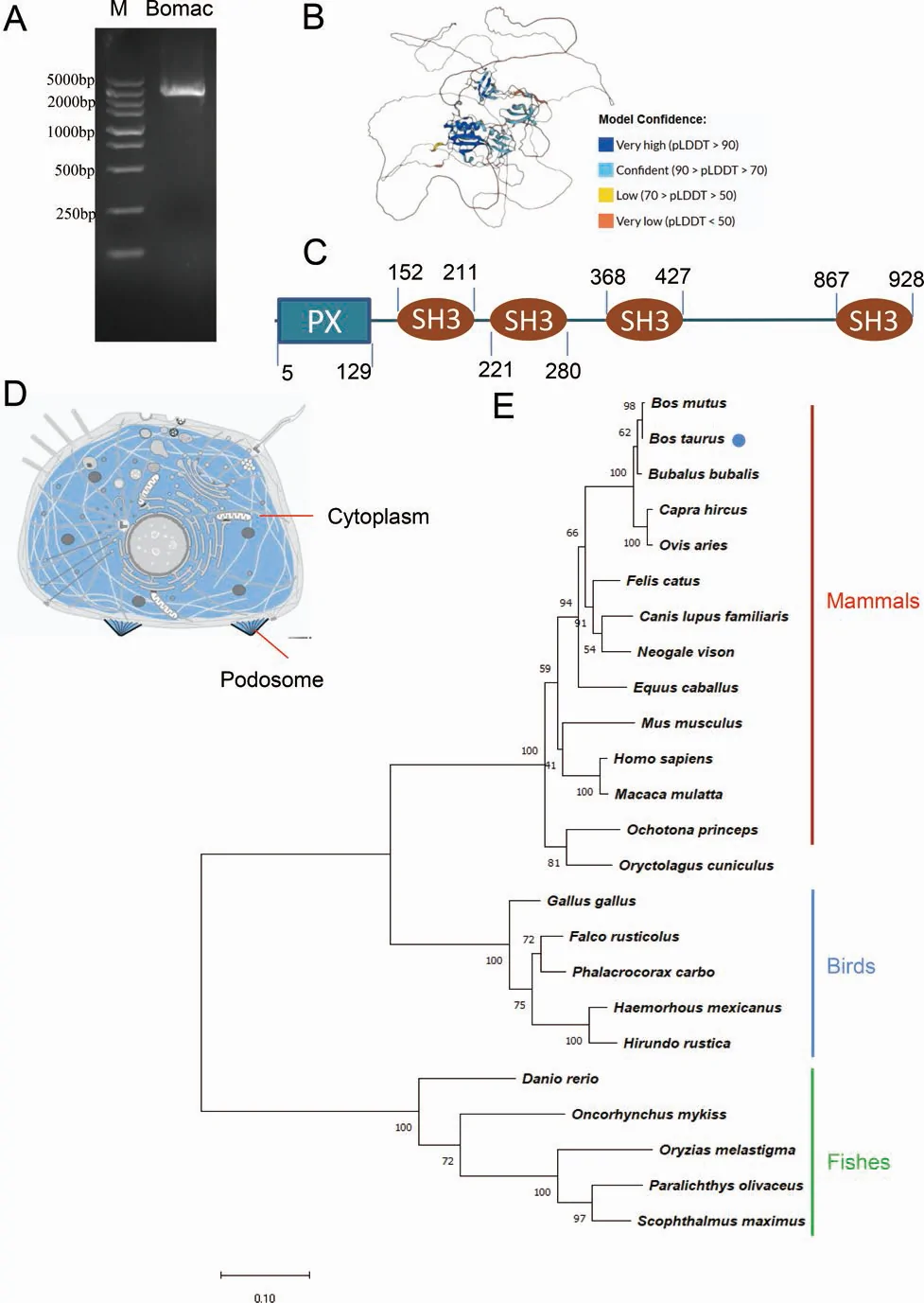
Sequence analyses of bovine SH3PXD2B. (**A**) Amplification of *SH3PXD2B* using Bomac DNA as a template. M: DL5000 Marker. (**B**) The structure of SH3PXD2B predicted by AlphaFold analysis (https://alphafold.ebi.ac.uk/). (**C**) The structural domain map of bovine SH3PXD2B referenced to UniProt (https://www.uniprot.org/). (**D**) The subcellular location of SH3PXD2B (https://www.uniprot.org). (**E**) A phylogenetic tree based on bovine SH3PXD2B between *Bos taurus* and other species. The phylogenetic tree was generated using MEGA-X. The sequences used were *Bos mutus*, XP_070213524.1; *Bos taurus*, XP_024837195.1; *Bubalus bubalis*, XP_025126633.3; *Canis lupus familiaris*, XP_038519280.1; *Capra hircus*, XP_017921047.1; *Danio rerio*, XP_005170345.1; *Equus caballus*, XP_070089019.1; *Falco rusticolus*, XP_037253440.1; *Felis catus*, XP_023115375.1; *Gallus gallus*, XP_025010804.2; *Haemorhous mexicanus*, XP_059715887.1; *Hirundo rustica*, XP_039934521.1; *Homo sapiens*, NP_001017995.1; *Macaca mulatta*, XP_028705753.1; *Mus musculus*, XP_006514775.1; *Neogale vison*, XP_044085415.1; *Ochotona princeps*, XP_004587480.2; *Oncorhynchus mykiss*, XP_021473168.2; *Oryctolagus cuniculus*, XP_051699577.2; *Oryzias melastigma*, XP_024118960.1; *Ovis aries*, XP_060256294.1; *Paralichthys olivaceus*, XP_019952883.2; *Phalacrocorax carbo*, XP_064314794.1; *Scophthalmus maximus*, XP_035480105.2.

Utilizing the cDNA of Bomac as a template, we successfully amplified the bovine SH3PXD2B gene (Fig. 4A). The amplified bovine SH3PXD2B gene is 2,787 base pairs in length and encodes a scaffold protein (928 amino acids) that exhibits 88.4% similarity to the human SH3PXD2B and 86.7% similarity to the mouse SH3PXD2B (Table 2). The domains of SH3PXD2B are highly conserved across different species (Table 2). SH3PXD2B is primarily localized in the cytoplasm and cytoskeleton (Fig. 4D). The protein contains four SH3 (Src Homology 3) domains and one PX (Phox Homology) domain (Fig. 4B and C). The SH3 domain is a protein interaction domain that can bind to proline-rich motifs (26), serving as a docking site for signaling molecules and mediating protein-protein interactions. For instance, the scaffolding protein N-WASP, which regulates the reorganization of the actin cytoskeleton, has been demonstrated to interact with the second SH3 domain of SH3PXD2B (27). ADAM15, a protein known to be associated with cell adhesion, was shown to interact with the fourth SH3 domain of the SH3PXD2B (28). The PX domain has been found to be associated with intracellular transport and localization. It has been determined that the primary function of the PX domain is to connect TKS scaffold proteins to the cell membrane by binding to phosphatidylinositol (18). A phylogenetic tree based on bovine SH3PXD2B between *Bos taurus* and other species was constructed, and three major branches were observed (Fig. 4E). Among them, *Bos mutus* showed the closest evolutionary relationship with bovine SH3PXD2B.

**TABLE 2:** Amino acid identity (%) of bovine SH3PXD2B and its domains (PX, SH3-1/2/3/4) compared to other species

Species	Full length	PX	SH3-1	SH3-2	SH3-3	SH3-4
Goat	96.9	99.2	100.0	100.0	100.0	98.4
Mouse	86.7	99.2	100.0	95.0	96.7	96.7
Human	88.4	100.0	98.3	96.7	100.0	93.4

### SH3PXD2B enhances phagocytosis in macrophages

Previous studies have shown that SH3PXD2B is associated with podosome structure and function. SH3PXD2B has been demonstrated to play a pivotal role in the process of actin cytoskeleton assembly and rearrangement (29). Furthermore, its absence has been shown to result in incomplete podosome assembly (30). Podosomes are dynamic, actin-rich membrane protrusions of certain cells (31). They can be formed by various cells, including endothelial cells, smooth muscle cells, osteoclasts, macrophages, and dendritic cells, and are involved in functions such as cell migration and phagocytosis (32–34).

**Fig 5:**
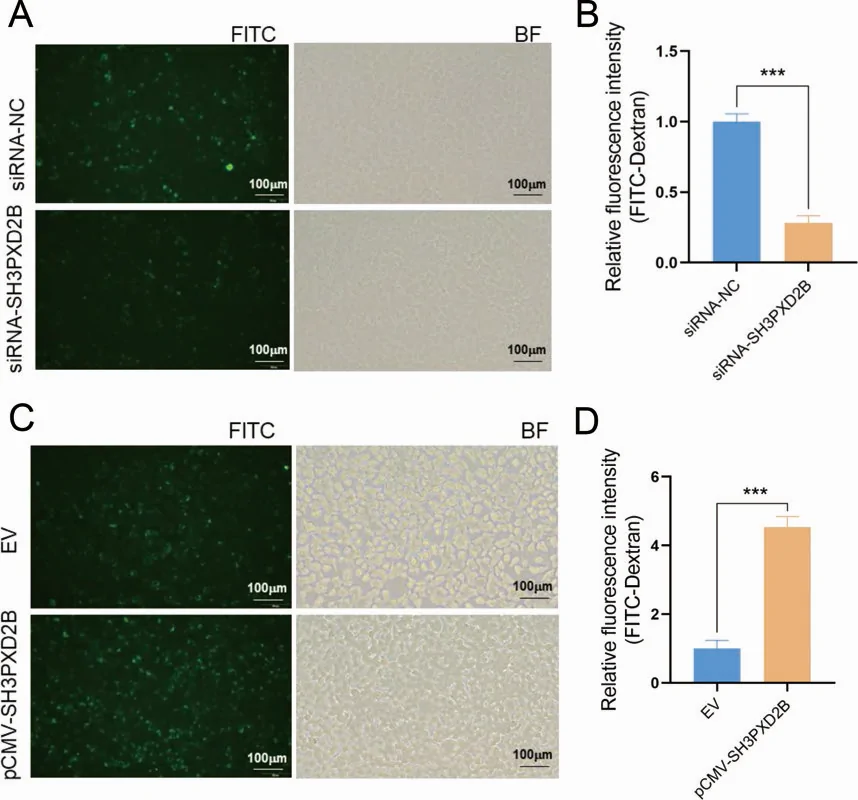
Bovine SH3PXD2B promotes phagocytosis. (**A**) FITC-Dextran levels of Bomac cells with SH3PXD2B knockdown. (**B**) The relative fluorescence intensity of FITC-Dextran internalized by Bomac with SH3PXD2B knockdown. (**C**) FITC-Dextran levels of Bomac cells with SH3PXD2B overexpression. (**D**) The relative fluorescence intensity of FITC-Dextran internalized by Bomac with SH3PXD2B overexpression.

**Fig 6:**
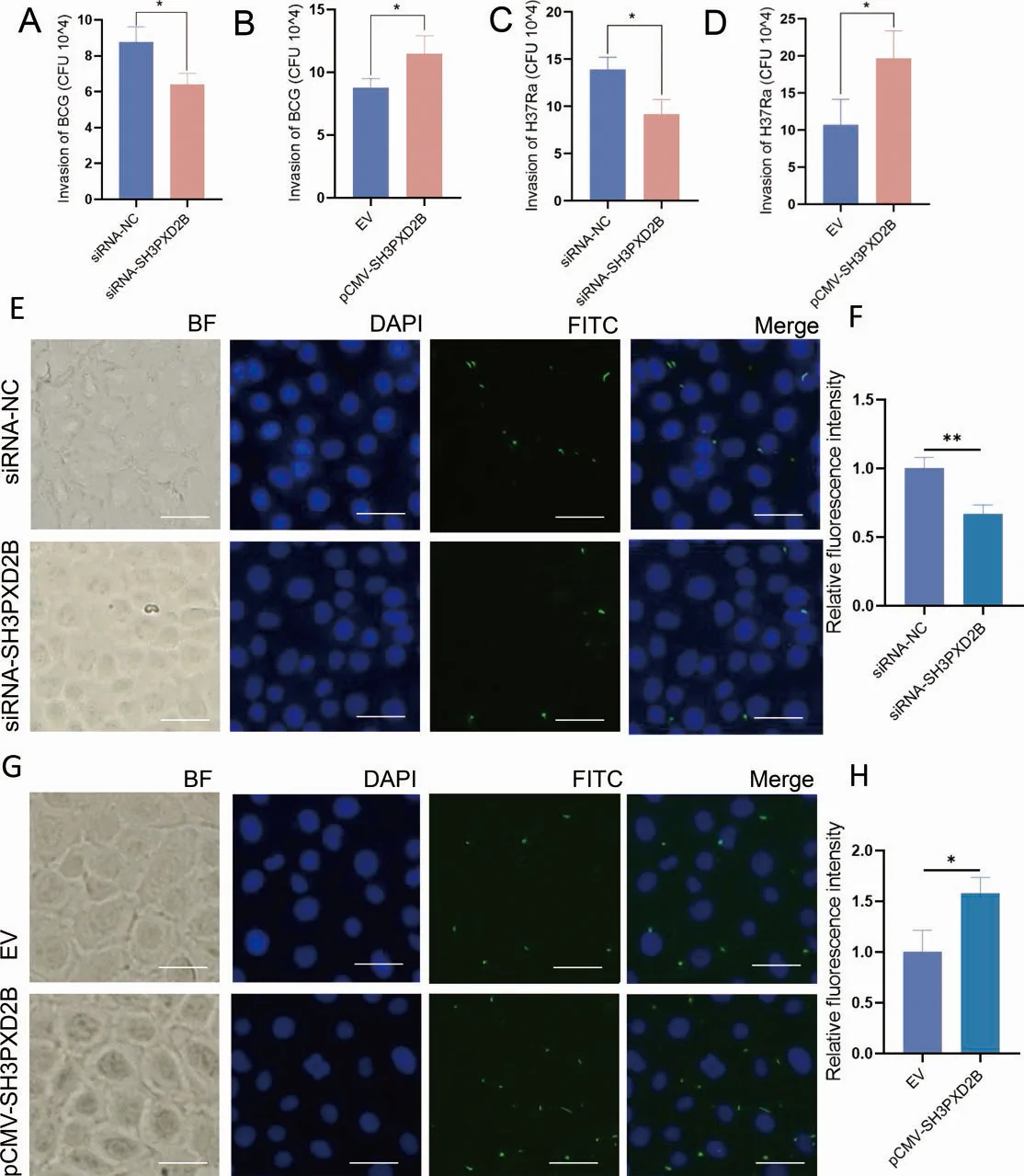
Bovine SH3PXD2B facilitates *M. bovis* BCG infection of Bomac. (**A**) Invasion of *M. bovis* BCG after infection of Bomac cells with SH3PXD2B knockdown. (**B**) Invasion of *M. bovis* BCG after infection of Bomac cells with SH3PXD2B overexpression. (**C**) Invasion of H37Ra after infection of Bomac cells with SH3PXD2B knockdown. (**D**) Invasion of H37Ra after infection of Bomac cells with SH3PXD2B overexpression. (**E**) FITC-BCG invades Bomac cells with SH3PXD2B knockdown. (**F**) Relative fluorescence intensity of FITC-BCG (SH3PXD2B knockdown). (**G**) FITC-BCG invades Bomac cells with SH3PXD2B overexpression. (**H**) Relative fluorescence intensity of FITC-BCG (SH3PXD2B overexpression).

In order to determine the role of SH3PXD2B in macrophage phagocytosis during mycobacterial infection, we used FITC-Dextran (1 mg/mL) to assess phagocytosis of Bomac cells with SH3PXD2B knockdown. The results of this study revealed that the suppression of SH3PXD2B expression led to a substantial decrease in the phagocytosis of dextran by the macrophages (Fig. 5A and B). Conversely, overexpression of SH3PXD2B resulted in a notable enhancement of phagocytosis compared to the control group (Fig. 5C and D). To determine whether these changes in phagocytosis affected BCG infection of Bomac cells, we counted the number of BCG invading Bomac after infection under SH3PXD2B knockdown and overexpression conditions. We found that knockdown of SH3PXD2B significantly reduced the number of BCG invading Bomac cells (Fig. 6A), while overexpression significantly increased the number of invading BCG cells (Fig. 6B). In a similar manner, experiments were conducted using Mtb H37Ra, and it was discovered that SH3PXD2B also contributes to H37Ra infection in Bomac (Fig. 6C and D). Subsequently, the bacterial invasion assay was conducted using FITC-BCG. Results show that the number of FITC-BCG in the SH3PXD2B knockdown group was less than that in the NC control group (Fig. 6E and F). The number of FITC-BCG in the SH3PXD2B overexpressed group cells is more than that in the EV group (Fig. 6G and H). These results suggest that following mycobacterial infection in Bomac, the upregulated expression of SH3PXD2B can promote mycobacterial infection by enhancing macrophage phagocytosis.

### SH3PXD2B inhibits macrophage migration

**Fig 7:**
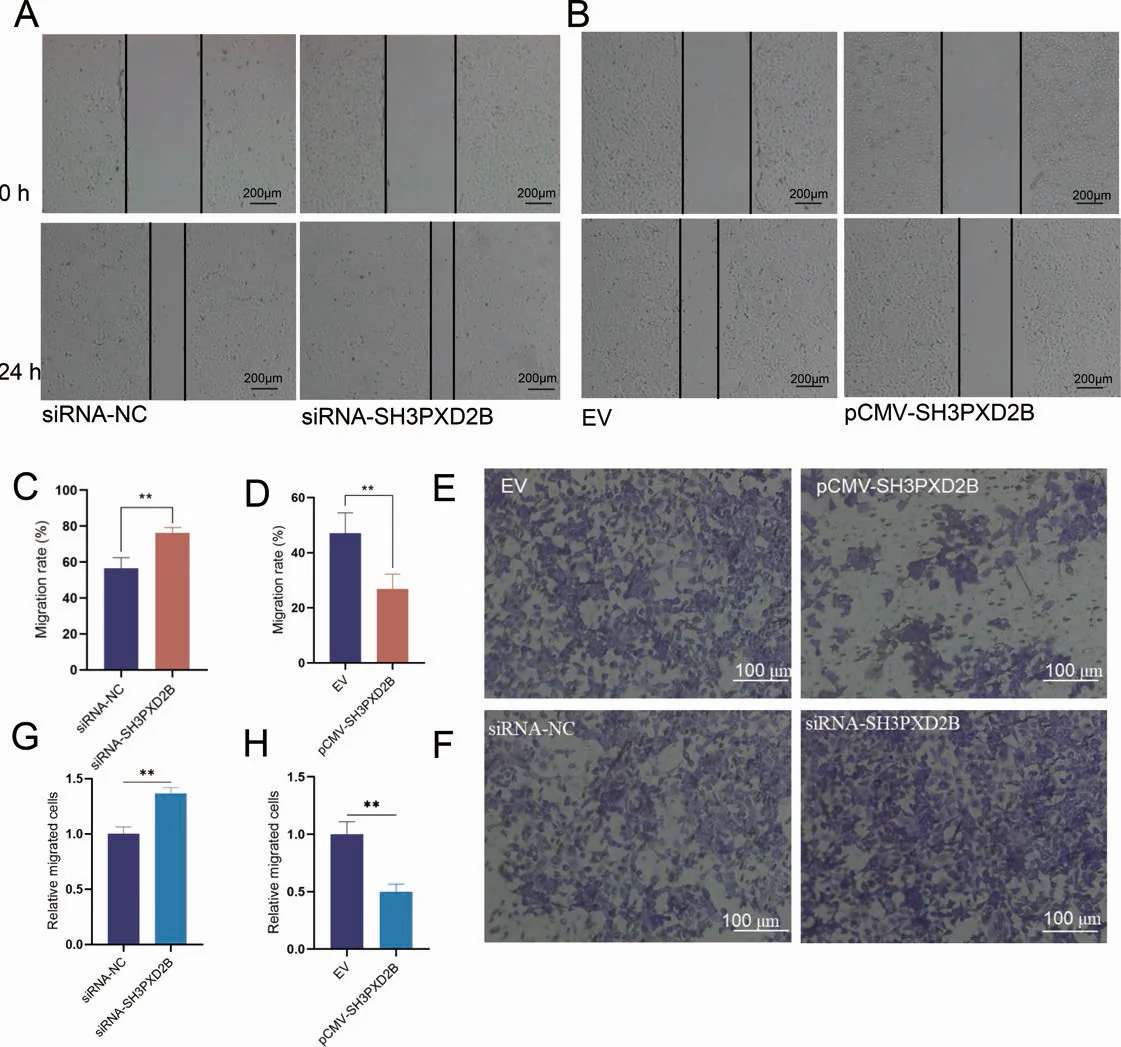
Bovine SH3PXD2B inhibits macrophage migration. (**A**) Cell migration rate of Bomac cells with SH3PXD2B knockdown. (**B**) Cell migration rate of Bomac cells with SH3PXD2B overexpression. (**C**) Cell migration rate of Bomac cells with SH3PXD2B knockdown. (**D**) Cell migration rate of Bomac cells with SH3PXD2B overexpression. (**E**) Transwell assay of Bomac cells with SH3PXD2B overexpression. (**F**) Transwell assay of Bomac cells with SH3PXD2B knockdown. (**G**) Relative migrated cells (SH3PXD2B knockdown). (**H**) Relative migrated cells (SH3PXD2B overexpression).

To evaluate whether SH3PXD2B affects the migration of Bomac cells, the scratch assay was performed to assess cell migration following SH3PXD2B knockdown or overexpression. The results showed that when siRNA was used to knock down SH3PXD2B, the average cell migration rate of Bomac cells in the NC control group was 56.58%; whereas that in the SH3PXD2B knockdown group was 76.28%, which was significantly higher than that in the NC control group (Fig. 7A and C). Further Transwell assay results revealed that the number of migrated Bomac cells in the SH3PXD2B knockdown group was significantly greater than that in the NC control group (Fig. 7F and G). The average cell migration rate in the EV group was 47.08%; whereas the average cell migration rate in the SH3PXD2B overexpression group was 26.85%, which was significantly lower than that in the empty vector control group (Fig. 7B and D). Meanwhile, the results of the Transwell assay also indicated that the number of Bomac cells successfully passing through the chamber in the SH3PXD2B overexpression group was significantly fewer than that in the EV group (Fig. 7E and H). These results indicated that SH3PXD2B overexpression reduced macrophage migration, while knockdown significantly enhanced migration.

### SH3PXD2B regulates macrophage phagocytosis and migration via MIF

**Fig 8:**
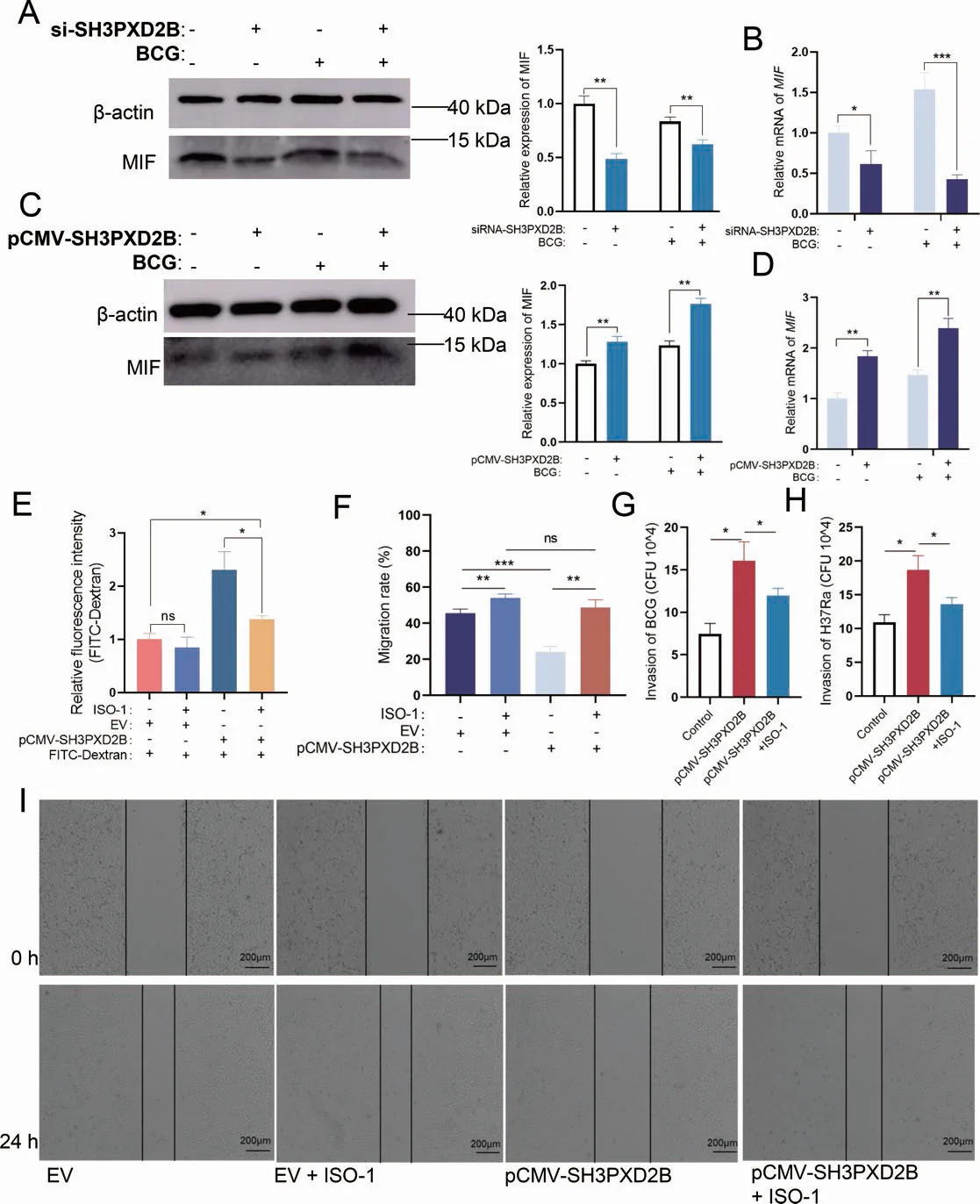
SH3PXD2B modulates cells migration and phagocytosis by regulating MIF. (**A**) Expression of MIF in Bomac cells with SH3PXD2B knockdown. (**B**) The mRNA levels of MIF in Bomac cells with SH3PXD2B knockdown. (**C**) Expression of MIF in Bomac cells with SH3PXD2B overexpression. (**D**) The mRNA levels of MIF in Bomac cells with SH3PXD2B overexpression. (**E**) The phagocytosis of Bomac cells treated with ISO-1. (**F**) Cell migration rate of Bomac cells treated with ISO-1. (**G**) The effect of ISO-1 on BCG infection in Bomac. (**H**) The effect of ISO-1 on H37Ra infection in Bomac. (**I**) Cell migration of Bomac cells treated with ISO-1.

A positive correlation between the expression levels of macrophage migration inhibitory factor (MIF; an inflammatory factor) and SH3PXD2B was found (Fig. 8A through D). MIF, a pleiotropic cytokine, has been shown to modulate the migration of macrophages and promote inflammatory responses (35, 36). In the present study, it was demonstrated that the overexpression of SH3PXD2B led to a significant inhibition in the migratory capacity of Bomac. However, when the MIF-specific inhibitor ISO-1 (20 μM) was administered (37), the migratory capacity of the Bomac was restored, and no statistically significant difference was observed in comparison to the control group (Fig. 8F and I). Similarly, after overexpressing macrophages, we treated Bomac cells with the MIF inhibitor for 2 h. Then we assessed the phagocytic capacity of cells using FITC-Dextran. Although the average fluorescence intensity of cells in the ISO-1 treatment group was significantly reduced compared to the untreated group when SH3PXD2B was overexpressed, it was still significantly higher than that in the EV control group under ISO-1 treatment (Fig. 8E). These indicate that the enhanced phagocytic ability of Bomac cells caused by SH3PXD2B overexpression can be partially restored by ISO-1. Furthermore, in the bacterial invasion assay, the intracellular CFU of BCG or H37Ra was found to be significantly reduced in the ISO-1-treated group in comparison to the SH3PXD2B overexpressing group (Fig. 8G and H). These results indicate that SH3PXD2B can modulate the expression of the MIF, thereby influencing the susceptibility of macrophages to mycobacteria.

## DISCUSSION

Our results unequivocally identify that the host SH3PXD2B is crucial in mediating mycobacteria invasion. The host protein SH3PXD2B is capable of regulating the phagocytic ability of macrophages. When SH3PXD2B is overexpressed, the phagocytic ability of Bomac cells is enhanced; whereas when SH3PXD2B is knocked down, the phagocytic ability of Bomac cells is weakened. Additionally, we found that when *M. bovis* BCG infects Bomac cells, the expression of SH3PXD2B in the cells is upregulated. This implies that *M. bovis* can actively enhance the phagocytic ability of macrophages during its infection process. Macrophages, as crucial innate immune cells in the body, play a vital role in defense through their phagocytic function, serving as a key pathway for capturing pathogenic microorganisms in the early stages of infection. Typically, macrophages employ various strategies to eliminate ingested pathogens (38). Pathogenic mycobacteria, however, paradoxically exploit this very mechanism, as successful entry into macrophages is a vital condition for acquiring immune protection and establishing early infection (3, 4). After entering macrophages, it can avoid being eliminated through various mechanisms, thus persisting in the macrophages. Therefore, enhancement of phagocytosis in macrophages through the regulation of SH3PXD2B after infection with *M. bovis* may be a survival strategy formed during its evolutionary process: that is, *M. bovis* may induce macrophages to be in a state of high phagocytic activity by actively upregulating the host protein SH3PXD2B, thereby facilitating the endocytosis of more *M. bovis* by macrophages, helping it invade host cells and obtain a safe ecological niche.

However, although our results have demonstrated that SH3PXD2B affects the phagocytic function of macrophages, there is still no more in-depth research on its related mechanisms and pathways. In this study, we found that the inhibition of the cytokine MIF can partially inhibit the phagocytic ability enhanced by the overexpression of SH3PXD2B, and the expression of MIF is regulated by SH3PXD2B, suggesting that MIF may be involved in the process of SH3PXD2B enhancing macrophage phagocytosis. Wu et al. (39) also found that MIF can promote the polarization of macrophages toward the M1 subtype, thereby enhancing bacterial phagocytosis. Therefore, MIF may be one of the mechanisms by which SH3PXD2B regulates cell phagocytosis.

Intriguingly, while SH3PXD2B promotes phagocytosis, it exerts a potent inhibitory effect on macrophage migration. This study showed that overexpression of SH3PXD2B inhibits the migration ability of macrophages, while knockdown of SH3PXD2B promotes cell migration. Similarly, Szeder et al. (40) found that the loss of SH3PXD2B protein can induce EMT-like changes in human colon cancer cells, thereby enhancing the migration and invasion abilities of the cells. *M. bovis* infection upregulates SH3PXD2B, thereby inhibiting cell migration, which may be a process aimed at retaining macrophages at the infection site. The retention of a large number of macrophages, neutrophils, and other cells at the infection site may affect the formation and stability of granuloma structures (41). Similarly, other studies have shown that reduced macrophage migration ability promotes infection with Mtb and enhances its pathogenicity: Berg et al. found that reduced macrophage migration leads to granuloma breakdown, increasing the severity of tuberculosis (42). However, some studies suggest that SH3PXD2B promotes cell migration, such as in hepatocellular carcinoma, melanoma, and oral squamous cell carcinoma (22–24). This discrepancy highlights the complexity of SH3PXD2B function and suggests that its effects may be specific in different cells. Given that SH3PXD2B is a scaffold protein with no known enzymatic activity, its diverse functions depend on the downstream signaling proteins it recruits (18, 43). Therefore, SH3PXD2B may be implicated in more intricate cellular signaling regulation under diverse conditions, thereby eliciting a variety of biological effects. The effect of the cytokine MIF on cell migration is highly similar to that of SH3PXD2B. In macrophages, MIF was named for its ability to inhibit macrophage migration. However, He et al. found that treatment with recombinant MIF can significantly enhance the aggressiveness of colorectal cancer (44). In our study, MIF was identified as a pivotal effector factor in this process. Despite the potential for MIF to be subject to regulation by multiple pathways (45), our findings suggest a positive correlation between the expression levels of the SH3PXD2B protein and MIF. Moreover, the use of MIF inhibitors resulted in the restoration of weakened macrophage migration caused by SH3PXD2B overexpression, and the number of BCG and H37Ra entering macrophages was significantly reduced. However, the exact signaling pathways connecting SH3PXD2B and MIF during *M. bovis* infection remain incompletely understood. The functional diversity exhibited by SH3PXD2B and MIF suggests that downstream mechanisms such as actin depolymerization may also be regulated by other factors. Therefore, the specific pathways and detailed regulatory mechanisms involved between the two need further research.

From the perspective of pathogen infection, we postulate that this SH3PXD2B-mediated enhanced uptake coupled with spatial confinement is a sophisticated pathogenic strategy orchestrated by mycobacteria. Whereby macrophages are recruited to the site of infection upon infection (3), some of the mycobacteria are engulfed by macrophages through phagocytosis. Then, mycobacteria regulate host factors such as SH3PXD2B, limiting macrophage movement and further promoting infection by enhancing phagocytosis. Furthermore, migrated weakened macrophages are unable to effectively phagocytose infected cells undergoing apoptosis, which can easily lead to secondary necrosis (42, 46).

It should be noted that the conclusions of this study are based on *in vitro* experiments using the Bomac cell line and the attenuated *M. bovis* BCG strain. BCG is an attenuated vaccine strain that does not fully reproduce the virulence or intracellular survival dynamics of pathogenic *M. bovis*. While some validation was performed with *M. tuberculosis* H37Ra, the use of attenuated strains may not fully reflect the interactions seen with highly virulent pathogens. Therefore, further validation in primary bovine macrophages and in animal models (bovine infected with virulent *M. bovis* strains) is essential to confirm the physiological relevance of our findings.

In summary, this study identified SH3PXD2B as a critical factor in the entry of mycobacteria into macrophages and the resulting cellular damage. Regulating SH3PXD2B to prevent large-scale mycobacterial invasion of cells may be an effective strategy for disease prevention or enhancing antibiotic efficacy. Consequently, SH3PXD2B is an important potential target for genetic breeding or HDT against tuberculosis.

## Data Availability

All sequences can be accessed directly through the NCBI nucleotide or protein databases using the provided accession numbers. Any other data will be made available upon reasonable request.

## SUPPLEMENTAL MATERIAL

Supplemental material aem.00675-26-s0001.docx Tables S1 and S2; Fig. S1 and S2.

ASM does not own the copyrights to Supplemental Material that may be linked to, or accessed through, an article. The authors have granted ASM a non-exclusive, world-wide license to publish the Supplemental Material files. Please contact the corresponding author directly for reuse.
