# Supplementary material for: SH3PXD2B is the critical host factor promoting the entry of mycobacteria
Source: Appl Environ Microbiol. 2026 Jul 17;92(8):e00675-26. doi: 10.1128/aem.00675-26 (PMC13488347; doi:10.1128/aem.00675-26)
Supplement: Supplementary file 1 [file aem.00675-26-s0001.docx]

Table S1. Primer sequences targeting positive hits (qPCR) used in this study

| Primer name | Sequence (5′-3′) |
| --- | --- |
| qLOC100300302-F | ACAACGCCCCTTGTTTCATT |
| qLOC100300302-R | GGCAAAGTGATGGTTCTGTCC |
| qPROX2-F | TGCCCGTAAAGATCCGCTTC |
| qPROX2-R | GGGAGCCTGATAGGGTTTGG |
| qLOC521656-F | CTCAAAAGGCAGCACCCAAA |
| qLOC521656-R | CAACCTGCTGAGGAACCAAGA |
| qSH3PXD2B-F | GCAATGGAAAACAGCTCGGG |
| qSH3PXD2B-R | GATCTCCTCGTAGCCTGCAC |
| qKCTD12-F | CCGACAGCACTCGTGGATTA |
| qKCTD12-R | ACGTTCAGCTCCACGATGTC |
| qMCIDAS-F | TGCTCCTCGTTTCTTGGGTC |
| qMCIDAS-R | GACGTCGCTAGGAGAGAACG |
| qLOC112444871-F | TGGGAGGTGACACTAAGCAAGA |
| qLOC112444871-R | GTTTGTCCCAACTGGGAGTCT |
| qpcr-CD59-F | TCTTCGTTAAGGCTGTGCCG |
| qpcr-CD59-R | GTGGAGCTCCTTCTCCCCTA |
| qpcr-GPR155-F | GCAGCATTGTGATAGCTGGC |
| qpcr-GPR155-R | GCACTGGACTGTCCTCGAAA |
| qpcr-COMMD10-F | GCTGTGTTACAGCTCGGAGT |
| qpcr-COMMD10-R | GGGAATCCAGCTGTGCTTGT |
| qpcr-M6PR-F | CACACCCTAGCGGACAACTT |
| qpcr-M6PR-R | CAGGCTGCTGTCCATCTCAA |
| qpcr-TMEM51-F | TCCAAGTTGTCCAAACGCCT |
| qpcr-TMEM51-R | GAGGGAGGAGGCACGTTTTT |
| qMGC152281-F | AGAGGGCCTTGATCTCACCT |
| qMGC152281-R | AAAGGTCTACCCGCCCCTAT |
| qNAB2-F | ATCCTATGCTCCCCCATTCCG |
| qNAB2-R | CCCTCATCCATCAGTGTCTGCT |
| qOTOGL-F | AACGTTGCATCTTGCGATGG |
| qOTOGL-R | AAGTTGCGCACCCCATTTTC |
| qLOC517475-F | CCATTCGAGACTGGCACACT |
| qLOC517475-R | TCCATTTCTGTGGGCTGCTT |
| qPAQR7-F | TGTGTTCACCGTGGATCGAG |
| qPAQR7-R | AACAGCGTGCGGAAGTAGAA |
| qFBXL5-F | TTCTCCGGCCTTTGCGTATT |
| qFBXL5-R | GCAGTCTAGTCCTTGGTGCT |
| qRCAN1-F | TAGCTCCCTGATTGCCTGTG |
| qRCAN1-R | GCTGCAGATAAGGGGTTGCT |
| qSLC39A4-F | GACCCGTCCTGTTCATCTGC |
| qSLC39A4-R | GGGCCGCCAGTGTATTTAAC |
| qCOX16-F | CGCTACGGAGTTCCCATGTT |
| qCOX16-R | CCAGTCATCAAAGGTGGAATCCT |

Table S1 (continued)

| Primer name | Sequence (5′-3′) |
| --- | --- |
| qC3H1orf226-F | TGCTGATTTCGTCACAGCCT |
| qC3H1orf226-R | AGATCAGGTACCGACACGGA |
| qFAM120A-F | CTTCATGAGTGGCGTGGACA |
| qFAM120A-R | GGTCGATGAGTGGGGTCTTT |
| qNEK7-F | GGCCTTACGACCGGATATGG |
| qNEK7-R | ACTCCATCCAAGAGACAGGC |
| qMap2k6-F | TCGACTCCGTGGCTAAAACC |
| qMap2k6-R | TCGTAGGGAAACCGGAGGAT |
| qGPRC6A-F | TCCAGAAGTGTGCCAGCTTT |
| qGPRC6A-R | GCCATTGCCACTGTGATGTC |
| qLOC100301263-F | GGCAGTGTCCGTGGTGATAA |
| qLOC100301263-R | CAGGCATTTCAGGGACTGGT |
| qTRAF3IP1-F | AAGACCCCAAAGCCGAAACA |
| qTRAF3IP1-R | ATCCAGGTACTACGGGGTCT |
| qIFI27L2-F | AGCTTTTGGGGCTTTGCTTG |
| qIFI27L2-R | CTGGCCCGAGTGGAGATTTT |
| qHMGCLL1-F | ACACGTATGGACAGGCTTCA |
| qHMGCLL1-R | TTGCATAAGGGCAACCACCT |

Table S2. The siRNAs used in this study

| siRNA name | Type | sequence (5'-3') |
| --- | --- | --- |
| siRNA-SH3PXD2B-1 | sense | CUGAAGAAGAAGAGAAGUATT |
|  | antisense | UACUUCUCUUCUUCUUCAGTT |
| siRNA-SH3PXD2B-2 | sense | GUAAGGAGGUGCUGAGGAATT |
|  | antisense | UUCCUCAGCACCUCCUUACTT |
| siRNA-SH3PXD2B-3 | sense | AGAGGAACAUGUUGGGAAATT |
|  | antisense | UUUCCCAACAUGUUCCUCUTT |
| siRNA-NC | sense | UUCUCCGAACGUGUCACGUTT |
|  | antisense | ACGUGACACGUUCGGAGAATT |

Figure S1 Establishment of Cas9 expressing Bomac cells. (A) Expression of Cas9 in Cas9 expressing Bomac cells. 1-7: Different monoclonal Bomac-Cas9 cell. 8: Wild-type Bomac. (B) Cell viability of different monoclonal Bomac-Cas9 cell.

Figure S2 Multiple sequence alignments of SH3PXD2B with their homologs. Multiple sequence alignments were performed using MEGA-X; the results were visualized using ESPript 3.0. The sequences used were: *Bos taurus*, XP_024837195.1; *Capra hircus*, XP_017921047.1; *Homo sapiens*, NP_001017995.1; *Mus musculus*, XP_006514775.1.
